# Effects of Dextran on the Gel Properties of Faba Bean Protein Isolates Prepared Using Different Processes

**DOI:** 10.3390/gels9120972

**Published:** 2023-12-12

**Authors:** Huihua Tang, Xinyi Li, Junfei Chen, Biqin Liu, Rong Tang, Yuchun Chen, Hong Li, Ling Zou, Qiao Shi

**Affiliations:** 1 Institute of Agro-Products Processing, Yunnan Academy of Agricultural Sciences, Kunming 65022, China; 2 College of Food Science and Technology, Yunnan Agricultural University, Kunming 650100, China; 3 Institute of Flower Research, Yunnan Academy of Agricultural Sciences, Kunming 65022, China

**Keywords:** commercial faba bean protein, faba bean protein prepared in-house, dextran, rheology, microstructure

## Abstract

The properties of faba bean (*Vicia faba* L.) protein isolate (FPI) gels depend on their starting protein material and can be modulated by the addition of polysaccharides. In order to investigate the interplay between these two factors, commercial FPI (FPI_1_) and FPI prepared in-house (FPI_2_) were used to fabricate glucono-delta-lactone-induced gels, with or without dextran (DX) addition. FPI_1_ exhibited lower solubility in water and a larger mean particle size, likely because it experienced extensive degradation due to the intense conditions involved in its preparation. The FPI_1_ gel showed a similar water-holding capacity as the FPI_2_ gel; however, its hardness was lower and viscoelasticity was higher. After DX addition, the hardness of both FPI gels decreased, while their water-holding capacity increased. Interestingly, DX addition decreased the viscoelasticity of the FPI_1_ gel but enhanced the viscoelasticity of the FPI_2_ gel. The microstructural analysis demonstrated that the density of the aggregation network decreased in the FPI_1_ gel after DX addition but increased in the FPI_2_ gel. This was consistent with the changes observed in the dominant protein interaction forces in these gels after DX addition. Overall, these findings have the potential to guide ingredient selection for the tailored preparation of FPI gels.

## 1. Introduction

The protein content of faba beans (*Vicia faba* L.) is as high as 25–30%, second only to that of soybean and much higher than that of rice, wheat, and other grains. Faba beans serve as important raw materials in a variety of food applications due to their rich starch and low fat content [1,2]. Wet extraction has proven to be the most effective method for extracting proteins from faba beans because it increases protein solubility [3]. However, processing equipment and parameters can also affect the physicochemical properties of protein isolates. Silva et al. confirmed that the protein isolates obtained from brewer’s spent grain via alkali extraction and acid precipitation under different processing parameters have different physicochemical and functional properties; moreover, these variations can affect their applications in food formulation [4]. 

Most studies on the functional properties of plant proteins use small-scale, laboratory extraction and freeze-drying procedures to prepare protein isolates [5,6]. However, there are large functional differences between the protein isolates prepared in the laboratory and those prepared through large-scale commercial processing. This is because commercial processing operations usually cause protein denaturation and aggregation, which can impair the functional properties of the protein [7]. Reports suggest that the difference between commercial soybean protein isolate (SPI) and laboratory samples is mainly a result of differences in protein aggregation and tertiary structure. Commercial SPI usually exhibits a high degree of denaturation and low solubility, which can weaken its functional properties during some food applications [8].

The addition of polysaccharides can improve the stability of plant protein gels [6]. Studies have shown that adding xanthan gum, konjac gum, inulin, and other polysaccharides can improve the rheological properties of SPI gels [9,10]. This phenomenon has also been observed in faba bean protein isolate (FPI) gels. The addition of starch and fiber enhances the structure of heat-induced FPI gels [5], while the addition of carrageenan significantly improves the mechanical properties of acid-induced FPI gels [11]. 

Dextran (DX) is a water-soluble, natural polysaccharide with α-(1→6) linkages and varied proportions of α-(1→2), α-(1→3), and α-(1→4) branched glycosidic linkages [12]. Some studies have examined the effects of adding α-(1→3)-branched DX on the rheological properties and microstructure of FPI [13,14]. However, the effects of DX on the properties of gels prepared using differentially processed FPIs remain to be explored. Moreover, so far, studies have not used any DX other than α-(1→3)-branched DX, nor induced gelation via means other than heat.

Glucono-delta-lactone (GDL) is often used to acidify proteins and subsequently fabricate acid gels. Hence, the aim of this study was to compare the properties of GDL-induced gels prepared from commercial FPI (FPI_1_) and FPI prepared in-house (FPI_2_), with or without the addition of α-(1→2)-branched DX. First, the two types of FPIs were characterized based on their solubility, sodium dodecyl sulfate–polyacrylamide gel electrophoresis (SDS-PAGE) profile, and particle size. Second, the physicochemical properties of the FPI gels were compared before and after DX addition through an analysis of texture, water-holding capacity (WHC), rheology, and interaction forces. Meanwhile, their microstructure was also evaluated using confocal laser scanning microscopy (CLSM). The goal of the study was to promote the adoption of FPIs and provide a reference for their application in polysaccharide/protein mixed gels in the food industry.

## 2. Results and Discussion

### 2.1. Characterization of FPIs

#### 2.1.1. Composition and Solubility

The composition of the FPIs is shown in Table 1. The protein contents of FPI_1_ and FPI_2_ were 80.70% and 77.00%, respectively. The FPIs had a fat content of 6.40–7.60% because the raw materials had not been defatted. The FPIs also showed differences in starch, ash, and dietary fiber content, which could be attributed to the use of different starting materials and processing equipment and parameters [15]. 

The gelation, emulsification, and foaming capacities of proteins are closely related to their solubility. Moreover, solubility can indicate the degree of protein denaturation [16]. As shown in Table 1, FPI_1_ exhibited a significantly lower solubility and higher turbidity than FPI_2_. Keivaninahr et al. found that commercial FPI produced via alkali extraction/isoelectric precipitation shows low solubility in water (8.3%) [17]. Further, the use of thermal treatment and organic solvents can cause high protein denaturation, resulting in low protein solubility and high turbidity [9]. Our findings, which indicate that commercial FPI preparation processes cause a relatively high degree of protein denaturation, are consistent with these reports.

**Figure 1:**
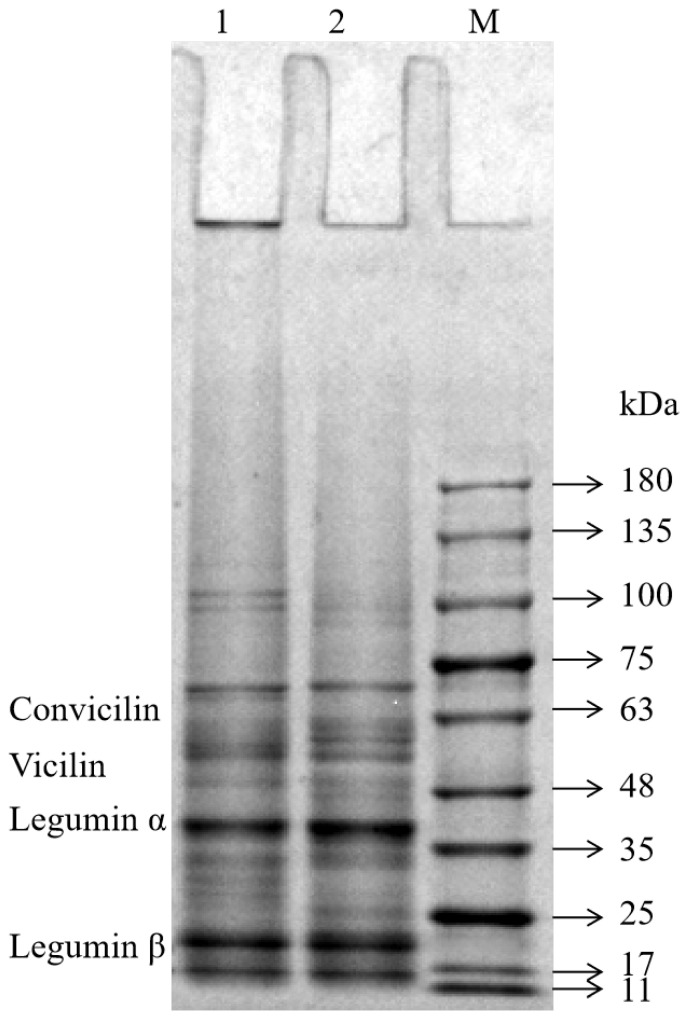
SDS-PAGE profiles of the FPIs under reduction conditions. Lane: 1-FPI_1_; 2-FPI_2_; M-protein markers.

The SDS-PAGE profiles of the FPIs, shown in Figure 1, were similar to those reported for other FPIs [15,18]. The FPIs contained various polypeptides with molecular weights ranging from 10 to 180 kilodaltons (kDa). The molecular weights of the convicilin fraction and vicilin fraction were 60–70 kDa and 46–55 kDa, respectively. Moreover, the two major legumin subunits were 38–40 kDa and 23 kDa in size [19]. The molecular weight distribution and band intensity of the two FPIs showed great similarity within the 11–75 kDa range. However, the intensity of the FPI_1_ bands was greater than that of the FPI_2_ bands in the range beyond 100 kDa. This suggested that FPI_1_ may contain large protein aggregates due to its higher degree of denaturation. Moreover, some proteins in the FPI_1_ fraction were too large to enter the gel and remained in the wells. Chao et al. also found that commercial pea protein prepared via heat treatment forms large protein aggregates (molecular weight > 200 kDa) that do not enter the gel (concentration, 10–15%) [20]. 

#### 2.1.2. Particle Size

**Figure 2:**
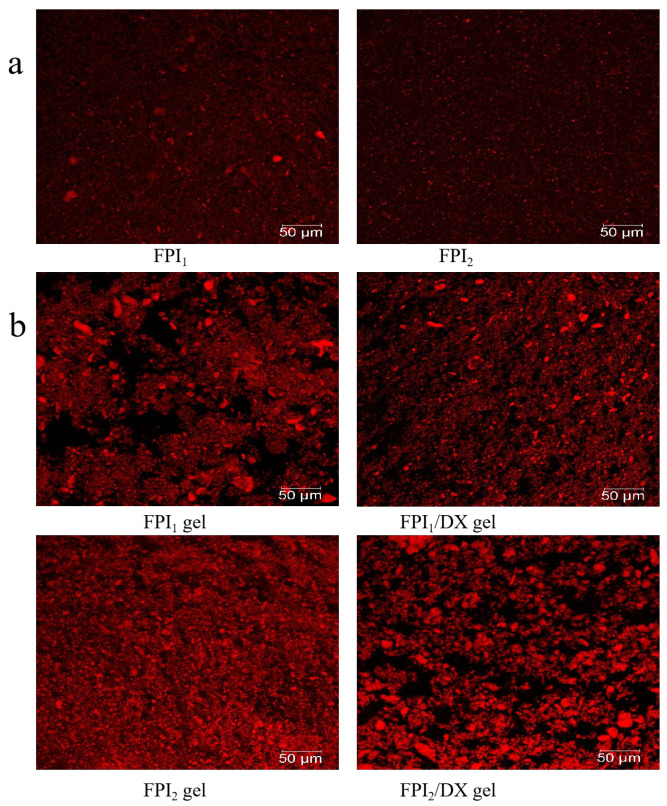
CLSM micrographs of the FPIs (**a**) and FPI/DX gels (**b**) (scale bar, 50 μm). Proteins, labeled with Rhodamine B, appear red, whereas water and DX appear black.

The mean particle size (D4,3) is related to the size and degree of the aggregation of protein particles. Additionally, it is a key determinant of gel properties [21]. The particle size distributions of FPI_1_ and FPI_2_ dispersions were significantly different (*p* < 0.05). As shown in Table 2 and Figure 2a, 50% of FPI_1_ and FPI_2_ particles were 37.45 μm and 21.64 μm in diameter, respectively. FPI_1_ also had a higher mean particle size (45.73 μm) than FPI_2_ (28.99 μm). In line with these findings, CLSM micrographs revealed that FPI_1_ particles exhibit a wide range of size distributions, whereas FPI_2_ particles appear to be more uniform and smaller in size. Vogelsang-O’Dwyer et al. found that the mean particle size of FPI produced by acid extraction/isoelectric precipitation is around 22.90 µm [15]. Our results demonstrated that FPI_1_ exhibits a higher degree of aggregation and a larger mean particle size. This may be because FPI_1_ undergoes greater denaturation due to prolonged exposure to high drying temperatures, which is characteristic of large-scale industrial production [7]. Hu et al. also demonstrated that in protein isolates, smaller particle sizes correspond to larger surface areas and increased protein–water interactions. Therefore, protein solubility and particle size are usually inversely proportional [22], consistent with the results of the present study. Collectively, the data on protein solubility and SDS-PAGE profiles indicated that FPI_1_ shows a higher degree of denaturation and larger aggregates than FPI_2_. Subsequently, the impact of DX supplementation on the properties of FPI gels prepared using the two FPIs was examined.

### 2.2. Characterization of Crude DX

**Figure 3:**
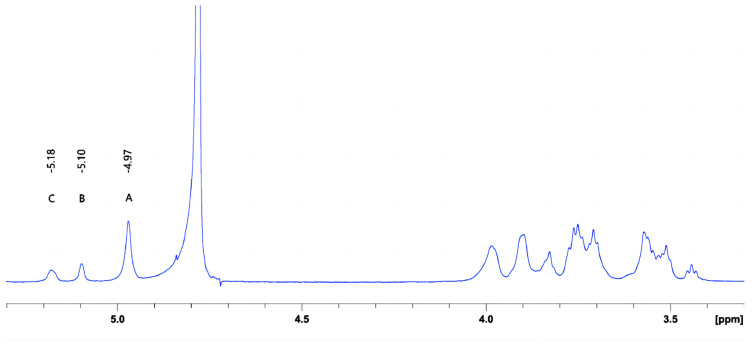
^1^H NMR spectrum of the DX obtained from *L. citreum* G26.

The characteristics of crude DX derived from *Leuconostoc citreum* strain G26 are shown in Table 3 and Figure 3. The weight-average molar mass of DX was 4.96 × 10^6^ Da. Based on the chemical shifts and intensities of anomeric proton signals, the peaks A, B, and C were assigned to the →6)-α-D-Glcp-(1→6) main-chain unit, the α-D-Glcp-(1→2) branch chain unit, and the →2,6)-α-D-Glcp-(1→6) main-chain unit, respectively [23]. The exopolysaccharide of *Leuconostoc citreum* strain G26 consisted of DX that primarily contained α-(1→6) glycosidic linkages (approximately 84.50%), with (1→2)-linked single-unit-branches (approximately 15.50%). The intrinsic viscosity of DX, a measure of the hydrodynamic volume of individual molecules in a solution (indicator of their stretching state and conformation) [24], was found to be 23.33 cm^3^/g. This value was lower than that (40 cm^3^/g) of DEX500 derived from *Leuconostoc mesenteroides* [25]. 

### 2.3. Preparation and Characterization of GDL-Induced FPI Gels

#### 2.3.1. Preparation of GDL-Induced FPI Gels

**Figure A1:**
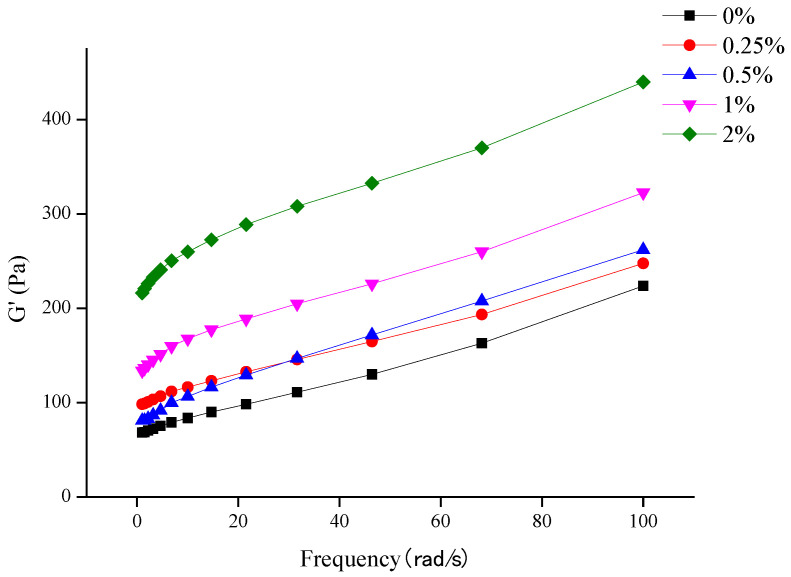
Effect of frequency on the dynamic rheological properties of DX/FPI gels prepared using different DX concentrations.

The fabrication of acid-induced gels can be divided into two stages. In the first stage, the protein is heated below the critical concentration, and then GDL is added to induce gelation. In this study, to prevent the formation of heat-induced gels, different FPI (6–12%, *w*/*v*) and GDL (0.5–2%, *w*/*v*) concentrations were tested. The optimal formulation was selected based on preliminary experiments to ensure that acid gels were generated, and not heat-induced gels. Visual examination revealed that self-supported gels were only formed when the FPI and GDL concentrations were at least 8% and 1%, respectively. Below these critical values, only viscous dispersions or non-supported gels were obtained. In our preliminary study, we compared the effects of DX concentrations (0.25, 0.5, 1, and 2%; *w*/*v*) on the stability of FPI gels. We found that 2% DX provided the best WHC (Table A1) and gel rigidity (storage modulus G’ analysis) (Figure A1) after storage for 48 h. Therefore, DX was added at a concentration of 2% to investigate its effects on FPI gels. 

#### 2.3.2. WHC and Hardness

As shown in Table 4, there was no significant difference in the WHC between the FPI_1_ and FPI_2_ gels (*p* > 0.05). However, the addition of DX improved the WHC of the mixed gels. Patole et al. found that the addition of 0.2 wt% locust bean gum markedly improves the WHC of quinoa protein isolate (QPI) gels. They hypothesized that the presence of locust bean gum facilitates the uptake of water and the formation of a compact network in QPI, resulting in a higher WHC [26]. Zhuang et al. also found that modified starch (0–2%), insoluble dietary fiber (0–2%), and konjac glucomannan (0–1%) can significantly improve the WHC of myofibrillar protein gels. This is likely because the polysaccharides are entrapped as fillers and promote the formation of a continuous gel matrix, modifying the distribution of the aqueous phase [27].

The hardness of the FPI_1_ gel was significantly lower than that of the FPI_2_ gel. This could be attributed to the differences in particle size and solubility between the two FPIs. Li et al. showed that an increase in protein solubility and a decrease in particle size result in larger protein–protein interactions and the formation of denser gel networks, thereby increasing gel hardness [28]. Interestingly, the addition of DX decreased the hardness of mixed gels. It has been reported that the addition of soybean insoluble dietary fiber increases the distance between soybean protein molecules, hindering protein cross-linking and generating a three-dimensional network with low gel hardness [29]. Jiang et al. found that starch hydrolysate compounds can interfere with the continuity of the network structure in tofu gels, thereby reducing their hardness [30]. In line with these studies, our findings show that the addition of specific polysaccharides can increase the WHC of gels and decrease their hardness.

#### 2.3.3. Dynamic Rheological Analysis

**Figure 4:**
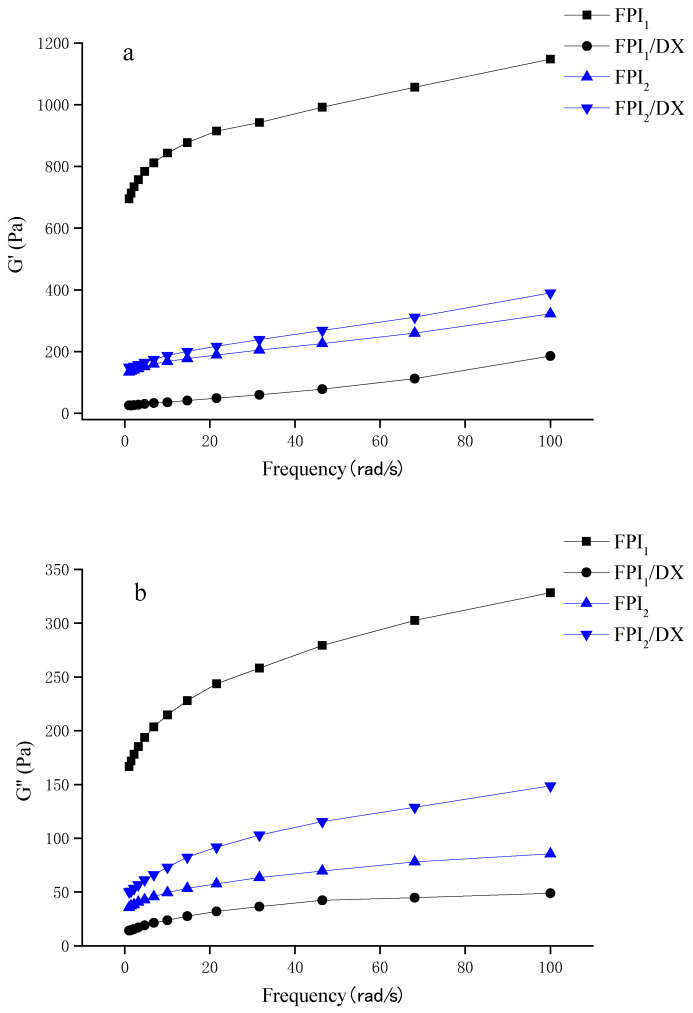
Storage modulus G’ (**a**) and loss modulus G" (**b**) of the FPI/DX gels, determined using a frequency sweep test.

The frequency dependence of the mixed gels is presented in Figure 4a,b. The G′ and G″ values of the gels increased in parallell as frequency increased, with the G′ values dominating over G″ across the whole frequency range. The FPI_1_ gel showed higher modulus values than the FPI_2_ gel. Generally, in commercial environments, proteins undergo denaturation during the isolation process. Denatured soy protein forms large aggregates, which act as precursors of the gel. Hence, pre-aggregation greatly improves the G’ value of a gel [31,32]. Shand et al. discovered that under the same gelation conditions, gels prepared using commercial pea protein exhibit a higher storage modulus than those prepared via laboratory-derived pea protein isolates. This difference can be ascribed to the different solubilities, conformations, and compositions of the protein isolates [33]. 

Interestingly, in this study, the effect of DX on the viscoelastic behavior of the FPI gels depended on the type of FPI. The addition of DX reduced the initially high storage modulus of the FPI_1_ gel. The addition of DX can interfere with the protein network of a gel by blocking interparticle aggregation, thus weakening the network structure and reducing the storage modulus of the gel [34]. Interestingly, we also found that the addition of DX improves the viscoelasticity of the FPI_2_ gel. Johansson et al. observed an increase in the G’ of FPI gels after the addition of starch/fiber. They believed that this was due to the adsorption of water by starch and fiber, which results in moisture stability and subsequently leads to a higher protein concentration in the surrounding matrix, improving the gel storage modulus [5].

#### 2.3.4. CLSM Observation

FPI gels with and without DX were also assessed using CLSM images, as shown in Figure 2b. The proteins, labeled with Rhodamine B, appear red, whereas water and DX appear black. The FPI_1_ gel had a non-uniform but more compact aggregation network than the FPI_2_ gel, likely due to the degree of protein denaturation and the presence of protein precipitates [35]. After DX addition, the polysaccharide became distributed within the gel skeleton, acting as a filler. In the FPI_1_ gel, DX supplementation resulted in the coagulation of protein aggregates forming smaller particles. Similarly, Çakır and Foegeding found that k-carrageenan can interfere with the crosslinking between aggregated whey protein isolate particles and interrupt the formation of a continuous whey protein isolate network [36]. 

In the FPI_2_ gel, phase separation was observed following DX addition, and the FPI_2_/DX gel showed denser protein aggregation. Monteiro et al. found that galactomannan addition causes pronounced phase separation in soybean protein gels, leading to increased viscoelasticity in soybean protein/galactomannan gels [6]. These results are consistent with the viscoelasticity results observed in the present study.

#### 2.3.5. Chemical Interactions and Free Sulfhydryl Groups

Table 5 shows the molecular forces between proteins and the free sulfhydryl groups in FPI gels. The FPI_1_ gel exhibited weaker hydrogen bonds and hydrophobic interactions than the FPI_2_ gel (*p* < 0.05), possibly because commercial FPI, with its larger particle size, shows lower interprotein interactions [37,38]. However, disulfide bonds were the dominant interaction forces in the FPI_1_ gel, and their content in FPI_1_ gels was significantly higher than that in FPI_2_ gels. One possible reason was that FPI_1_ initially had more exposed SH– groups due to protein denaturation during processing, which facilitated the formation of disulfide bonds. After DX addition, the content of free SH– in the FPI_1_/DX gel increased significantly, but the content of disulfide bonds showed a significant decrease. This could be attributed to the DX-induced inhibition of disulfide bond formation, consistent with the findings reported by Li et al. for Adlay starch/gluten protein gels [39]. 

In contrast, after DX addition, the dominant interaction forces in FPI_2_ gels shifted from disulfide bonds to hydrophobic interactions. This increase in hydrophobic interactions in the FPI_2_/DX gel may be due to the phase separation effect, which is in line with the denser FPI_2_ aggregation observed during microstructural analysis. Similar to these findings, a study by Xu et al. also showed that the hydrophobic interactions in the emulsion gel system are significantly enhanced after the addition of the neutral polysaccharide inulin [40].

## 3. Conclusions

In this study, commercial FPI (FPI_1_) and FPI prepared in-house (FPI_2_) were used to prepare acid-induced gels in the presence or absence of DX to investigate the influence of DX on the textural, rheological, and microstructural properties of FPI gels. FPI_1_ had a higher average particle size (45.73 μm) than FPI_2_ (28.99 μm), and the solubility of FPI_1_ (17.29%) was lower than that of FPI_2_ (66.53%). FPI_1_ alone formed gels with lower gel hardness and higher gel moduli. Moreover, DX addition increased the WHC and decreased the hardness of all gels. However, the effect of DX on gel viscoelasticity was dependent on the properties of the starting protein, with DX addition decreasing the gel viscoelasticity of the FPI_1_ gel while enhancing that of the FPI_2_ gel. CLSM images proved that DX addition had opposite effects on the density of the protein network in these gels, which could explain the distinct viscoelasticity changes observed in the mixed gels. We believe that the degree of protein denaturation during commercial and laboratory FPI preparation can affect the aggregation behavior of the proteins and their interaction with DX, thus influencing the properties of FPI gels. Nevertheless, it should be noted that these differences could also partly result from the different sources of faba beans. Overall, this study adds to the current knowledge of FPI gelation and could guide the application of FPIs in protein-based foods. However, the mechanisms underlying the effects of temperature, solvent treatment, and other factors involved in the preparation process on the gel properties of FPIs need to be delineated.

## 4. Materials and Methods

### 4.1. Materials

FPI_1_ was purchased from Baoji Senrui Biological Chemical Co., Ltd. (Baoji, China). Dried faba bean (*Vicia faba* L.) was procured from the Food Crops Research Institute, Yunnan Academy of Agricultural Sciences. *L. citreum* G26 was preserved in the Food Microbial Culture Collection, Yunnan Academy of Agricultural Sciences. A BCA Protein Assay Kit (PC0020), Precast-GLgel Hepes SDS-PAGE 4–15% (C621104-0001), 2× SDS-PAGE loading buffer (C508319-0001), and biological semipermeable membranes (14-kDa molecular weight cut-off) were purchased from Sangon Biotech Co., Ltd. (Shanghai, China). The BiosPMTM Rainbow Protein Marker (11–180 kDa) was purchased from Beijing Solaibao Technology Co., Ltd. (Beijing, China). Chemicals of analytical grade were purchased from Sinopharm Chemical Reagent Co., Ltd. (Shanghai, China).

### 4.2. FPI Preparation 

According to the information provided by the supplier, FPI_1_ was produced using the alkali extraction/isoelectric precipitation method. The protein dispersion was flash sterilized and then spray-dried, thereby producing a dried powder.

FPI_2_ was prepared from faba beans using the alkali (1 M NaOH, pH 9.5) extraction/isoelectric precipitation method described by Makri et al. [41]. In brief, flour was dispersed in deionized water (1:10) and the pH was subsequently adjusted to 9.5 with NaOH. Then, the mixture was stirred for 40 min at room temperature. Following centrifugation at 7000× *g* for 15 min, the supernatant was collected. The pellet was washed with distilled water (1:5), and after centrifugation at 7000× *g* for 15 min, the supernatant was collected and combined with the supernatant collected earlier. The pH of this mixture was adjusted to 4.5. The protein precipitate was recovered by centrifugation at 7000× *g* for 15 min. Its pH was adjusted to 7.0, and the isolate was vacuum lyophilized for subsequent analysis.

#### 4.2.1. Compositional Analysis

Compositional analysis to measure moisture, protein, ash, and starch content was performed using the AOAC 925.09, AOAC 992.15, AOAC 923.03, and AOAC 996.11 methodology, respectively. Fat content was measured using the EPA 9071B-hexane extraction method. Dietary fiber content was determined based on the Chinese Standard GB 5009.10-2003 method [42].

#### 4.2.2. Protein Solubility and Turbidity Measurement

FPI powders were dissolved in deionized water (5 mg/mL) by stirring for 2 h and then centrifuged at 7000× *g* and 4 °C for 15 min. The protein concentration in the supernatant, expressed as a percentage of the total protein, was determined using a BCA Protein Assay Kit. The turbidity of FPI dispersions (5 mg/mL) was measured using a Microplate Reader (Multiskan GO, Thermo, Waltham, MA, USA) based on the absorbance at 600 nm [43].

#### 4.2.3. SDS-PAGE 

FPI powder (10 mg) was suspended in 2 mL deionized water, and 2 mL SDS-PAGE loading buffer was then added to this suspension. The mixture was boiled for 5 min and then centrifuged at 7000× *g* and 4 °C for 5 min. The supernatant (10 μL, 2 mg/mL) was loaded onto an SDS-PAGE gel. Electrophoresis was carried out at 110 V for 80 min. Subsequently, the gel was stained with Coomassie Brilliant Blue R-250 overnight and then destained until the bands became clear [17]. The blots were analyzed using Molecular Imager Gel Doc XR+ with Image labTM (Bio-Rad Laboratories).

#### 4.2.4. Particle Size Distribution

The particle sizes of the FPI dispersions (2%, *w*/*v*) were measured with a particle size analyzer based on laser diffraction (PSA1190, Anton Paar, Graz, Austria). The protein refractive index was set at 1.45, the absorption parameter was 0.01, and the dispersant (water) refractive index was 1.33. The mean diameter (D4,3) and the size distributions D10, D50, and D90 were recorded for further analysis.

### 4.3. Crude DX Preparation

Crude DX was isolated from an *L. citreum* G26 fermentation culture. The *L. citreum* G26 strain was activated through static incubation in MRS liquid medium at 37 °C for two generations. The seed solution was inoculated at a ratio of 1% (*v*/*v*) into the MRS liquid medium, which only contained sucrose (10%) as the carbon source. It was then incubated at 30 °C for 48 h. The culture was centrifuged at 7000× *g* and 4 °C for 10 min. Subsequently, the supernatant was treated with trichloroacetic acid at a final concentration of 5%, and the protein was removed after magnetic stirring for 2 h and centrifugation at 7000× *g* and 4 °C for 10 min. The resulting supernatant was precipitated with a triple volume of ethanol for 12 h. The pellet was collected after centrifugation at 7000× *g* for 10 min and dissolved in deionized water. The solution was dialyzed using a biological semipermeable membrane for 72 h, and the deionized water was changed every 12 h. After dialysis, the retentate was freeze-dried to obtain DX. 

### 4.4. Effect of DX on FPI Gel Characteristics

#### 4.4.1. Preparation of GDL-Induced Gels

Initially, different final FPI (6, 8, 10, and 12% *w*/*v*) and GDL concentrations (0, 0.5, 1%, and 2 *w*/*v*) were tested to select the best formulation for the FPI gels. The selection was based on visual examination [44]. Subsequently, 8% FPI and 1% GDL were selected to ensure good gel stability. Then, different concentrations of DX from *L. citreum* G26 (0.25, 0.5, 1, and 2% *w*/*v*) were tested to select the optimal concentration for FPI/DX gels. Finally, 2% DX addition was selected as the optimal parameter.

The FPI/DX gels were prepared using a previously described method [45]. Briefly, FPI (8%, *w*/*v*) was dispersed thoroughly in 10 mL of distilled water at 25 °C by stirring for 2 h. This was followed by the addition of DX powder (2%, *w*/*v*) and stirring at 4 °C overnight to obtain an FPI/DX dispersion, which was incubated at 80 °C for 30 min. Then, the dispersion was charged with 1% *w*/*v* GDL and stirred for 2 min. After cooling to room temperature, the mixture was placed at 4 °C overnight to obtain the FPI/DX mixed gel. Some FPI/DX mixed gel samples were vacuum lyophilized for subsequent analysis. 

#### 4.4.2. WHC Analysis

Fresh gel samples (10 g) were centrifuged at 7000× *g* for 10 min. The WHC was expressed as the ratio of gel mass after decanting to the gel mass before centrifugation.

#### 4.4.3. Hardness Analysis

Gel hardness was examined utilizing a texture analyzer (TMS-Touch, FTC, Sterling, VA, USA) equipped with a cylindrical probe (*p*/0.5R). Gel hardness was determined by applying a constant compression force of 0.045 N at a constant speed of 2.0 mm/s to a compression depth of 4 mm. Hardness was defined as the peak force required for the first compression. 

#### 4.4.4. Dynamic Oscillatory Rheology

The frequency sweeps of the FPI/DX gels were measured using a HAAKE rotation rheometer (Thermo Fisher, Waltham, MA, USA). A pp50 plate with a plate spacing of 1 mm was used. The frequency (f) range was 1–100 rad/s and the strain was 1% within the linear viscoelastic range. The storage modulus (G’) and loss modulus (G") of the FPI gels as a function of (f) were measured. 

#### 4.4.5. CLSM

First, for FPI samples, 0.001% (*w*/*v*) Rhodamine B was mixed with the FPI dispersion (8%). A small volume of this sample was transferred to a glass slide. For gel samples, the mixed dispersion was stained with 0.001% (*w*/*v*) Rhodamine B prior to heat treatment and the addition of GDL. Afterward, a small volume of the mixture was transferred onto a glass slide and incubated at 4 °C for 24 h to allow gel formation. A confocal microscope (Leica TCS SP8, Wetzlar, Germany) was employed to observe the FPI samples and gels using an excitation wavelength of 514 nm and an emission wavelength range of 551–655 nm, at 400× magnification.

#### 4.4.6. Analysis of Chemical Interactions in the Gel

Various solvents were employed to assess protein solubility with the aim of elucidating the molecular mechanisms governing gel formation [46]. The solvents used for the dissolution of freeze-dried samples at a concentration of 20 mg/mL were as follows: 0.05 mol/L NaCl (LA), 0.6 mol/L NaCl (LB), 0.6 mol/L NaCl + 1.5 mol/L urea (LC), 0.6 mol/L NaCl + 8 mol/L urea (LD), and 0.6 mol/L NaCl + 8 mol/L urea + 0.5 mol/L 2-mercaptoethanol (LE). The dispersions were mixed at 5 °C for 1 h and centrifugated at 7000× *g* for 15 min. The protein concentration in the supernatant was determined using the Bradford method and expressed as g soluble protein/L of solution. The differences in solubility between LA and LB, LB and LC, LC and LD, and LD and LE indicated the content of ionic bonds, hydrogen bonds, hydrophobic interactions, and disulfide bonds, respectively. 

#### 4.4.7. Measurement of Free SH– Content

The content of free SH– groups in the FPI/DX gels was determined using Ellman’s reagent (DTNB) [47]. In summary, freeze-dried samples (30 mg) were weighed and then suspended in a Tris-glycine buffer (0.086 M Tris, 0.09 M glycine, 4 mM EDTA; pH 8.0; 10.0 mL, Sinopharm Chemical Reagent Co., Ltd. (Shanghai, China)) with 0.1 mL DTNB (4 mg/mL). The mixture was incubated in the dark for 1 h under constant stirring and then centrifuged at 7000× *g* and 4 °C for 10 min. The absorbance of the supernatant was determined at 412 nm against a reagent blank.

μmol SH–/g = 73.53 × A412 × D/C(1)

Here, D represents the dilution coefficient (which was “1” for the present study), and C (mg/mL) represents the protein concentration.

### 4.5. Statistical Analysis

Data are presented as the mean ± standard deviation (SD). All experiments were performed in triplicate. SPSS 22.0 (IBM Inc., Armonk, NY, USA) was used for variance analysis. The level of significance (LSD) was preset at *p* < 0.05. Origin 8.5 (Origin Lab Inc., Northampton, MA, USA) was used to plot all graphs.

## Figures and Tables

**Table 1:** Composition, solubility, and dispersion turbidity (T) of the FPIs.

Protein samples	Protein (%)	Starch (%)	Fat (%)	Ash (%)	Dietary fiber (%)	Moisture (%)	Solubility (%)	T (A_600_)
FPI_1_	80.70	2.60	7.60	3.80	0.60	6.42	17.29	0.77
FPI_2_	77.00	2.60	6.40	5.20	0.20	8.32	66.53	0.39

**Table 2:** Particle size parameters of the FPIs.

Protein Samples	D10 (μm)	D50 (μm)	D90 (μm)	D4,3 (μm)
FPI_1_	13.63 ± 0.04 ^a^	37.45 ± 0.19 ^a^	82.97 ± 0.49 ^a^	45.73 ± 0.23 ^a^
FPI_2_	8.36 ± 0.04 ^b^	21.64 ± 0.05 ^b^	55.99 ± 0.11 ^b^	8.99 ± 0.17 ^b^

D10, D50, and D90 represent the diameters through which 10%, 50%, and 90% of the particles pass, respectively. D4,3 represents the mean particle size. Different superscript letters in the same column represent significant differences (*p* < 0.05).

**Table 3:** Source microorganism, weight-average molar mass (Mw), intrinsic viscosity, and glucopyranosyl unit composition of the crude dextran sample.

DX Sample	Source Microorganism	M_w_ (g/mol)	Intrinsic Viscosity (cm^3^/g)	Constituent Glucopyranosyl Units	Distribution (%)
G26	*L. citreum* G26	4.98 × 10^6^	23.33	(A) →6)-α-D-Glcp-(1→6)-	68.56
				(B) α-D-Glcp-(1→2)-	15.50
				(C) →2,6)-α-D-Glcp-(1→6)-	15.94

(A–C) represent different types of glucopyranosyl units, identified based on their anomeric proton signals (Figure 3).

**Table 4:** Effect of DX isolated from *L. citreum* G26 on the water-holding capacity (WHC) and hardness of FPI/DX gels.

Gel Samples	WHC (%)	Hardness (N)
FPI_1_	62.72 ± 0.54 ^c^	0.16 ± 0.0076 ^b^
FPI_1_/DX	68.09 ± 1.45 ^b^	0.11 ± 0.0040 ^c^
FPI_2_	61.23 ± 1.26 ^c^	0.21 ± 0.0046 ^a^
FPI_2_/DX	72.33 ± 2.27 ^a^	0.18 ± 0.0220 ^b^

Different superscript letters (a–c) in the same column represent significant differences (*p* < 0.05).

**Table 5:** Intermolecular forces and free sulfhydryl groups (free SH–) in FPI gels.

Gel Samples	Intermolecular Interaction Forces				Free SH– (μmol/g)
	Ionic Bonds (g/L)	Hydrogen Bonds (g/L)	Hydrophobic Interactions (g/L)	Disulfide Bonds (g/L)	
FPI_1_	0.12 ± 0.00 ^cC^	0.07 ± 0.02 ^cC^	1.42 ± 0.03 ^cB^	4.70 ± 0.12 ^aA^	8.80 ± 0.20 ^d^
FPI_1_/DX	0.02 ± 0.00 ^dC^	0.13 ± 0.00 ^cC^	1.11 ± 0.03 ^cB^	2.87 ± 0.12 ^bA^	11.66 ± 0.21 ^c^
FPI_2_	0.85 ± 0.02 ^aC^	2.2 ± 0.10 ^aB^	2.27 ± 0.09 ^bB^	2.99 ± 0.02 ^bA^	13.61 ± 0.13 ^b^
FPI_2_/DX	0.26 ± 0.05 ^bD^	1.2 ± 0.04 ^bC^	3.44 ± 0.16 ^aA^	2.88 ± 0.10 ^bB^	20.01 ± 0.12 ^a^

Different lowercase superscript letters (a–d) in the same column represent significant differences (*p* < 0.05). Different uppercase superscript letters (A–D) in the same row represent significant differences (*p* < 0.05).

## Data Availability

The data presented in this study are openly available in article.

## Appendix

**Table A1:** Effect of DX concentration on the water-holding capacity (WHC) of the DX/FPI mixed gels.

Concentration (%)	WHC (%)
0	61.23 ± 1.26 ^d^
0.25	62.79 ± 0.48 ^d^
0.5	66.45 ± 0.51 ^c^
1	70.15 ± 0.67 ^b^
2	72.33 ± 2.27 ^a^

Different superscript letters (a–d) represent significant differences (*p* < 0.05).

## References

[1] Schmelter L., Rohm H., Struck S. (2021). Gluten-Free Bakery Products: Cookies Made from Different Vicia Faba Bean Varieties. Futur. Foods.

[2] Gangola M.P., Ramadoss B.R., Jaiswal S., Chan C., Mollard R., Fabek H., Tulbek M., Jones P., Sanchez-Hernandez D., Anderson G.H. (2021). Faba Bean Meal, Starch or Protein Fortification of Durum Wheat Pasta Differentially Influence Noodle Composition, Starch Structure and in Vitro Digestibility. Food Chem..

[3] Hewage A., Olatunde O.O., Nimalaratne C., Malalgoda M., Aluko R.E., Bandara N. (2022). Novel Extraction Technologies for Developing Plant Protein Ingredients with Improved Functionality. Trends Food Sci. Technol..

[4] da Silva A.M.M., Almeida F.S., da Silva M.F., Goldbeck R., Sato A.C.K. (2023). How Do PH and Temperature Influence Extraction Yield, Physicochemical, Functional, and Rheological Characteristics of Brewer Spent Grain Protein Concentrates?. Food Bioprod. Process..

[5] Johansson M., Johansson D., Ström A., Rydén J., Nilsson K., Karlsson J., Moriana R., Langton M. (2022). Effect of Starch and Fibre on Faba Bean Protein Gel Characteristics. Food Hydrocoll..

[6] Monteiro S.R., Rebelo S., da Cruz e Silva O.A.B., Lopes-da-Silva J.A. (2013). The Influence of Galactomannans with Different Amount of Galactose Side Chains on the Gelation of Soy Proteins at Neutral PH. Food Hydrocoll..

[7] Ma K.K., Greis M., Lu J., Nolden A.A., McClements D.J., Kinchla A.J. (2022). Functional Performance of Plant Proteins. Foods.

[8] Verfaillie D., Janssen F., Van Royen G., Wouters A.G.B. (2023). A Systematic Study of the Impact of the Isoelectric Precipitation Process on the Physical Properties and Protein Composition of Soy Protein Isolates. Food Res. Int..

[9] Lopes-da-Silva J.A., Monteiro S.R. (2019). Gelling and Emulsifying Properties of Soy Protein Hydrolysates in the Presence of a Neutral Polysaccharide. Food Chem..

[10] Tseng Y.C., Xiong Y.L. (2009). Effect of Inulin on the Rheological Properties of Silken Tofu Coagulated with Glucono-δ-Lactone. J. Food Eng..

[11] Dille M.J., Knutsen S.H., Draget K.I. (2022). Gels and Gelled Emulsions Prepared by Acid-Induced Gelation of Mixtures of Faba Bean (Vicia Faba) Protein Concentrate and λ-Carrageenan. Appl. Food Res..

[12] Chen Z., Tian Y., Zhang W., Guang C., Meng X., Mu W. (2019). Novel Dextransucrase Gtf-DSM, Highly Similar in Sequence to Reuteransucrase GtfO, Displays Unique Product Specificity. J. Agric. Food Chem..

[13] Xu Y., Pitkänen L., Maina N.H., Coda R., Katina K., Tenkanen M. (2018). Interactions between Fava Bean Protein and Dextrans Produced by *Leuconostoc Pseudomesenteroides* DSM 20193 and *Weissella Cibaria* Sj 1b. Carbohydr. Polym..

[14] Dong H., Li Y., Jia C., Zhang B., Niu M., Zhao S., Xu Y. (2022). Mechanism behind the Rheological Property Improvement of Fava Bean Protein by the Presence of Dextran. Food Hydrocoll..

[15] Vogelsang-O’Dwyer M., Petersen I.L., Joehnke M.S., Sørensen J.C., Bez J., Detzel A., Busch M., Krueger M., O’Mahony J.A., Arendt E.K. (2020). Comparison of Faba Bean Protein Ingredients Produced Using Dry Fractionation and Isoelectric Precipitation: Techno-Functional, Nutritional and Environmental Performance. Foods.

[16] Yang X., Ke C., Li L. (2021). Physicochemical, Rheological and Digestive Characteristics of Soy Protein Isolate Gel Induced by Lactic Acid Bacteria. J. Food Eng..

[17] Keivaninahr F., Gadkari P., Zoroufchi Benis K., Tulbek M., Ghosh S. (2021). Prediction of Emulsification Behaviour of Pea and Faba Bean Protein Concentrates and Isolates from Structure-Functionality Analysis. RSC Adv..

[18] Bühler J.M., Dekkers B.L., Bruins M.E., Van Der Goot A.J. (2020). Modifying Faba Bean Protein Concentrate Using Dry Heat to Increase Water Holding Capacity. Foods.

[19] Warsame A.O., O’Sullivan D.M., Tosi P. (2018). Seed Storage Proteins of Faba Bean (Vicia Faba L): Current Status and Prospects for Genetic Improvement. J. Agric. Food Chem..

[20] Chao D., Aluko R.E. (2018). Modification of the Structural, Emulsifying, and Foaming Properties of an Isolated Pea Protein by Thermal Pretreatment. CYTA-J. Food.

[21] Shen X., Zhao C., Guo M. (2017). Effects of High Intensity Ultrasound on Acid-Induced Gelation Properties of Whey Protein Gel. Ultrason. Sonochem..

[22] Hu H., Cheung I.W.Y., Pan S., Li-Chan E.C.Y. (2015). Effect of High Intensity Ultrasound on Physicochemical and Functional Properties of Aggregated Soybean β-Conglycinin and Glycinin. Food Hydrocoll..

[23] Yang Y., Peng Q., Guo Y., Han Y., Xiao H., Zhou Z. (2015). Isolation and Characterization of Dextran Produced by Leuconostoc Citreum NM105 from Manchurian Sauerkraut. Carbohydr. Polym..

[24] Li X., Liu Y., Li N., Xie D., Yu J., Wang F., Wang J. (2016). Studies of Phase Separation in Soluble Rice Protein/Different Polysaccharides Mixed Systems. LWT-Food Sci. Technol..

[25] Mende S., Peter M., Bartels K., Dong T., Rohm H., Jaros D. (2013). Concentration Dependent Effects of Dextran on the Physical Properties of Acid Milk Gels. Carbohydr. Polym..

[26] Patole S., Cheng L., Yang Z. (2022). Impact of Incorporations of Various Polysaccharides on Rheological and Microstructural Characteristics of Heat-Induced Quinoa Protein Isolate Gels. Food Biophys..

[27] Zhuang X., Jiang X., Zhou H., Chen Y., Zhao Y., Yang H., Zhou G. (2020). Insight into the Mechanism of Physicochemical Influence by Three Polysaccharides on Myofibrillar Protein Gelation. Carbohydr. Polym..

[28] Li J., Wu M., Wang Y., Li K., Du J., Bai Y. (2020). Effect of PH-Shifting Treatment on Structural and Heat Induced Gel Properties of Peanut Protein Isolate. Food Chem..

[29] Priyashantha H., Quintáns A.P., Baixauli R., Vidanarachchi J.K. (2019). Type of Starter Culture Influences on Structural and Sensorial Properties of Low Protein Fermented Gels. J. Texture Stud..

[30] Jiang Z.Q., Wang J., Stoddard F., Salovaara H., Sontag-Strohm T. (2020). Preparation and Characterization of Emulsion Gels from Whole Faba Bean Flour. Foods.

[31] Cramp G.L., Kwanyuen P., Daubert C.R. (2008). Molecular Interactions and Functionality of a Cold-Gelling Soy Protein Isolate. J. Food Sci..

[32] Wang X., Yu M., Wang Z., Luo K., Adhikari B., Miao S., Liu S. (2022). Modulation of Soy Protein Isolate Gel Properties by a Novel “Two-Step” Gelation Process: Effects of Pre-Aggregation with Different Divalent Sulfates. Food Chem..

[33] Shand P.J., Ya H., Pietrasik Z., Wanasundara P.K.J.P.D. (2007). Physicochemical and Textural Properties of Heat-Induced Pea Protein Isolate Gels. Food Chem..

[34] Lazaridou A., Vaikousi H., Biliaderis C.G. (2008). Impact of Mixed-Linkage (1→3, 1→4) β-Glucans on Physical Properties of Acid-Set Skim Milk Gels. Int. Dairy J..

[35] Klost M., Brzeski C., Drusch S. (2020). Effect of Protein Aggregation on Rheological Properties of Pea Protein Gels. Food Hydrocoll..

[36] Çakir E., Foegeding E.A. (2011). Combining Protein Micro-Phase Separation and Protein-Polysaccharide Segregative Phase Separation to Produce Gel Structures. Food Hydrocoll..

[37] Zhao H., Li W., Qin F., Chen J. (2016). Calcium Sulphate-Induced Soya Bean Protein Tofu-Type Gels: Influence of Denaturation and Particle Size. Int. J. Food Sci. Technol..

[38] Tang C.H., Wang X.Y., Yang X.Q., Li L. (2009). Formation of Soluble Aggregates from Insoluble Commercial Soy Protein Isolate by Means of Ultrasonic Treatment and Their Gelling Properties. J. Food Eng..

[39] Li C., Chen G., Ran C.X., Liu L., Wang S., Xu Y., Tan Y., Kan J. (2019). Adlay Starch-Gluten Composite Gel: Effects of Adlay Starch on Rheological and Structural Properties of Gluten Gel to Molecular and Physico-Chemical Characteristics. Food Chem..

[40] Xu Q., Qi B., Han L., Wang D., Zhang S., Jiang L., Xie F., Li Y. (2021). Study on the Gel Properties, Interactions, and PH Stability of Pea Protein Isolate Emulsion Gels as Influenced by Inulin. LWT.

[41] Makri E.A., Papalamprou E.M., Doxastakis G.I. (2006). Textural Properties of Legume Protein Isolate and Polysaccharide Gels Eleousa. J. Sci. Food Agric..

[42] (2003). Determination of Crude Fiber in Vegetable Foods. General Administration of Quality Supervision.

[43] Huang K., Shi J., Li M., Sun R., Guan W., Cao H., Guan X., Zhang Y. (2022). Intervention of Microwave Irradiation on Structure and Quality Characteristics of Quinoa Protein Aggregates. Food Hydrocoll..

[44] Brito-Oliveira T.C., Bispo M., Moraes I.C.F., Campanella O.H., Pinho S.C. (2017). Stability of Curcumin Encapsulated in Solid Lipid Microparticles Incorporated in Cold-Set Emulsion Filled Gels of Soy Protein Isolate and Xanthan Gum. Food Res. Int..

[45] Tang H., Chen J., Liu B., Wang X., Shi Q., Li H. (2022). Rheological Properties of Dextran/Faba Bean Protein Composite Gel. Mod. Food Sci. Technol..

[46] Gómez-Guillén M.C., Borderías A.J., Montero P. (1997). Chemical Interactions of Nonmuscle Proteins in the Network of Sardine (Sardina Pilchardus) Muscle Gels. LWT-Food Sci. Technol..

[47] Beveridge T., Tome S.J., Nakai S. (1974). Determination of SH- and SS-Groups in Some Food Proteins Using Ellman’s Reagent. J. Food Sci..

